# A rare case of neuroleptic malignant syndrome presenting with serious hyperthermia treated with a non-invasive cooling device: a case report

**DOI:** 10.4076/1752-1947-3-6170

**Published:** 2009-02-19

**Authors:** Christian Storm, Rolf Gebker, Anne Krüger, Lutz Nibbe, Joerg C Schefold, Frank Martens, Dietrich Hasper

**Affiliations:** 1 Department of Nephrology and Medical Intensive Care, Charité - Campus Virchow, Universitätsmedizin Berlin, Germany; 2 Berlin Heart Centre, Department of Cardiology, Berlin, Germany

## Abstract

**Introduction:**

A rare side effect of antipsychotic medication is neuroleptic malignant syndrome, mainly characterized by hyperthermia, altered mental state, haemodynamic dysregulation, elevated serum creatine kinase and rigor. There may be multi-organ dysfunction including renal and hepatic failure as well as serious rhabdomyolysis, acute respiratory distress syndrome and disseminated intravascular coagulation. The prevalence of neuroleptic malignant syndrome is between 0.02% and 2.44% for patients taking neuroleptics and it is not necessary to fulfil all cardinal features characterizing the syndrome to be diagnosed with neuroleptic malignant syndrome. Because of other different life-threatening diseases matching the various clinical findings, the correct diagnosis can sometimes be hard to make. A special problem of intensive care treatment is the management of severe hyperthermia. Lowering of body temperature, however, may be a major clinical problem because hyperthermia in neuroleptic malignant syndrome is typically unresponsive to antipyretic agents while manual cooling proves difficult due to peripheral vasoconstriction.

**Case presentation:**

A 22-year-old Caucasian man was admitted unconscious with a body temperature of 42°C, elevated serum creatine phosphokinase, tachycardia and hypotonic blood pressure. In addition to intensive care standard therapy for coma and shock, a non-invasive cooling device (Arctic Sun 2000^®^, Medivance Inc., USA), originally designed to induce mild therapeutic hypothermia in patients after cardiopulmonary resuscitation, was used to lower body temperature. After successful treatment it became possible to obtain information from the patient about his recent ambulant treatment with Olanzapin (Zyprexa®) for schizophrenia.

**Conclusion:**

Numerous case reports have been published about patients who developed neuroleptic malignant syndrome due to Olanzapin (Zyprexa®) medication. Frequently hyperthermia has been observed in these cases with varying outcomes. In our case the only residual impairment for the patient is dysarthria with corresponding symmetric cerebellar pyramidal cell destruction demonstrated by increased signal intensity in T2-weighted magnetic resonance imaging, most likely caused by the excessive hyperthermia.

## Introduction

The common application of atypical neuroleptics such as Olanzapin (Zyprexa®) is the treatment of schizophrenia or severe bipolar disorders. Neuroleptic malignant syndrome (NMS) is an uncommon side effect of neuroleptics, independent of the dosage and duration of drug therapy [1,2]. The main clinical findings are hyperthermia, altered mental state, haemodynamic dysregulation, elevated serum creatine kinase and rigor, but not all findings must necessarily occur together [2,3]. Intensive care therapy is necessary because of the life-threatening symptoms. Treatment mainly involves withdrawal of the causative agent and supportive care. Rapid lowering of body temperature is necessary once it reaches 40°C or above, since prognosis is not only related to maximum body temperature but also to duration of hyperthermia. It is reported that hyperthermia can cause brain lesions, especially in the cerebellum, although the mechanism is not fully clarified [2,4]-[6]. Furthermore, there is a known discrepancy between the measured central (rectal, oesophageal) temperature and the brain temperature which is often higher. Therefore, there is a need for aggressive manual cooling with cold blankets and ice water although efficacy of these measures may be limited by cutaneous vasoconstriction, which diminishes the capacity for heat loss. Therefore, in some cases extracorporeal cooling is necessary. In this case presentation, the Arctic Sun 2000^®^ cooling device was used to treat the patient's hyperthermia. To our knowledge this is the first time Arctic Sun 2000^®^ has been used in a case of NMS for the treatment of excessive hyperthermia.

## Case presentation

A 22-year-old male Caucasian patient was admitted to our intensive care unit (ICU) after he was found on the street in a condition of fluctuating awareness with aggressive behaviour changing to somnolence. He had low blood pressure, tachycardia and laboured respiration. Because of his fluctuating awareness it was impossible to obtain any history from the patient when he was brought to the ICU by the rescue service. On admission, the patient had a Glasgow coma scale (GCS) of six and because of respiratory insufficiency, intubation and mechanical ventilation was immediately necessary. Only intravenous agents were used for sedation (Fentanyl, Midazolam and Etomidate). Furthermore, he was haemodynamically unstable with a shock index of two. He presented with mild muscle rigidity and an excessive, elevated body temperature of 42°C. He carried no personal documents and there was no possibility of identifying the patient or of acquiring any information about his prior medical history. Because antipyretic drugs had no effect on the severe state of hyperthermia we quickly decided to use the non-invasive Arctic Sun 2000^®^ cooling device (Arctic Sun 2000^®^, Medivance Inc., USA) in order to lower his dangerous temperature.

**Figure 1:**
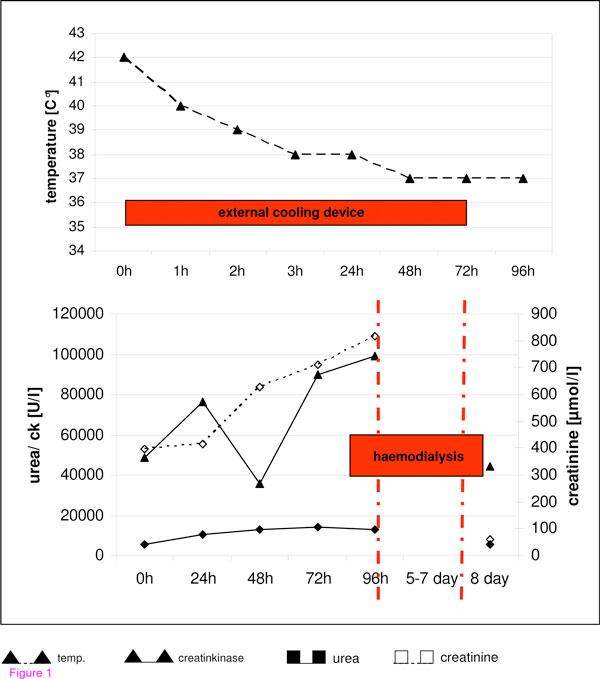
**Time course of ICU-time**. Course of central body temperature, urea and creatinine is shown. Time course of cooling therapy and haemodialysis is shown in addition as a red bar.

This system originally was designed to induce mild therapeutic hypothermia in patients after cardiopulmonary resuscitation. The water flushed energy transfer pads were pasted onto the patient's skin and the Arctic Sun 2000^®^ was used in the manual mode by controlling the flushed water temperature. The heat transfer and cooling performance of the system is induced by direct conduction and approximates the performance of water immersion by providing high energy transfer. The water temperature inside the energy transfer pads was set to 10°C and the target temperature of 38.5°C was reached after 120 minutes. For online temperature monitoring computer based monitoring software was used. Adjunctive drug therapy during the cooling therapy was sedation with a benzodiazepine (Midazolam) and an opioid (Fentanyl); however muscle relaxants were not administered. The patient presented initially only with mild muscle rigidity and had no tremor. Thus, we decided to start aggressive symptomatic therapy. Dantrolene was discussed as a further possible option in case symptomatic therapy did not improve the patient's condition. The laboratory results on admission are given in Table 1. A toxicological screening test was performed on his blood and urine, showing in both only a cannabis metabolite and the benzodiazepine used for sedation. Because of hyperthermia of unknown origin, a cerebral computed tomography (CT) scan and a lumbar puncture were carried out to exclude, on the one hand, any cause of infection and, on the other hand, a cerebral cause for decreased awareness and autonomic dysregulation. The cerebral CT scan and the cerebrospinal fluid analysis showed no pathology explaining the patient's condition. Because of massive rhabdomyolysis, the patient rapidly developed acute renal failure with a need for haemodialysis. An interesting observation was that the hyperthermia was not influenced by any antipyretic drug given to the patient when the use of the cooling device was shortly interrupted. Although the cause of hyperthermia was still unclear, the general condition of the patient improved and the respirator therapy was discontinued on the third day after admission. In the course of time, he was able to give detailed information about his schizophrenia and had been treated as an outpatient with Olanzapine (Zyprexa®) 10 mg daily by his psychiatrist. With regard to the initial clinical findings this neuroleptic drug treatment was the most likely cause of his hyperthermia. He was discharged from ICU and, because of improving renal function, haemodialysis was stopped. The only residual impairment was dysarthria which he declared was new after the phase of hyperthermia. A magnetic resonance image (MRI) scan showed symmetric multiple contrast agent enhancement in the cerebellum and in one small spot in the frontal lobe (see Figure 1). It is hypothesized that these lesions are direct pyramidal cell lesions caused by hyperthermia, as described in the literature in other cases [4,5,7].

**Table 1:** Laboratory results on admission

Value	Result	Range	Unit
Lactatdehydrogenase	2020	[<80]	U/l
Aspartat-aminotransferase	785	[<50]	U/l
Alanin-aminotransferase	157	[<50]	U/l
pH	7.27	7.35-7.45	
pCO2	42	32-46	mmHg
pO2	610	72-100	mmHg
HCO3-	19	21-26	mmol/l
SBE	-8	-2/+3	mmol/l
Lactate	5.8	0.55-2.2	mmol/l
Haemoglobin	18.7	12-18	g/dl
Glucose	6.9	<5.5	mg/dl
Sodium	148	135-150	mmol/l
Potassium	4.5	3.5-5	mmol/l
ScvO2	59	>70%	%

Values in square brackets are normal values.

**Figure 2:**
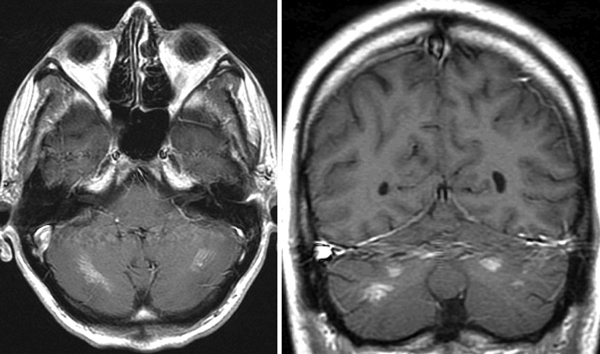
**MRI scan (axial and coronar) with symmetric contrast agent enhancement in the cerebellum (white spots)**.

## Discussion

A case presentation of neuroleptic malignant in a young, male patient with known schizophrenia and treated with Olanzapin is reported. This syndrome is well described in the literature, especially with one of its main key symptoms of excessive hyperthermia. Our patient showed the cardinal symptoms of NMS [1]-[3,6,8]. Most other possible causes of hyperthermia were ruled out with a negative toxicological screening (except cannabis), normal cerebral CT scan, normal results of liquor analysis, or were all very unlikely in this case (see Table 2). Therefore, it was supposed that the patient's ambulant neuroleptic medication was the most possible cause for the NMS. The most important differential diagnoses with their most common triggers are shown in Table 2. The various common findings of all of them include elevation of body temperature, muscle rigidity with corresponding elevation of serum creatine kinase levels, excessive perspiration, haemodynamic instability and fluctuating awareness. Because of these similar clinical findings, the patient's history with the initial trigger may be extremely helpful in establishing the correct diagnosis. In this case presentation the history of the ambulant neuroleptic drug medication was initially unknown and therefore it was not possible to establish a definitive diagnosis on admission. In contrast to patients with an elevated hypothalamic set point in cytokine induced fever, hyperthermia patients do not respond to or benefit from any antipyretic drug therapy [9]. Since clinical and laboratory findings did not suggest an infectious cause of hyperthermia in our patient, it was decided to cool down the patient with a device especially designed to bring about hypothermia in order to not only achieve rapid, but also controlled, temperature decrease. Arctic Sun 2000^®^ is a non-invasive cooling device used primarily on patients after cardiopulmonary resuscitation to induce mild therapeutic hypothermia, according to the International Liaison Committee on Resuscitation (ILCOR) recommendations [10]. The Arctic Sun 2000^®^ energy transfer pads cover approximately 40% of the patient's total body surface area and temperature controlled water circulates at a high flow rate inside the pads. The effect achieved by the system is very similar to water immersion and results in highly efficient heat exchange between the patient and the energy transfer pads. Although it has been reported that in some cases with hyperthermia after Olanzapin (Zyprexa®) medication temperature has normalized on its own after a length of time, but from our point of view for a patient with a body temperature higher than 40°C, a conservative treatment was not indicated. We therefore decided to bring the patient's temperature from 42°C to 38.5°C as quickly as possible to avoid damage caused by such a hyperthermic condition [9]. The initial cooling phase from 42°C down to 38.5°C took 120 minutes. To hold the temperature at 38.5°C and to avoid a further temperature decrease, the water temperature inside the energy transfer pads was increased from 10°C to 30°C. With these settings it was possible to stabilize the patient's temperature between 37 to 38°C. No serious side effects, such as cardiac arrhythmia or additional haemodynamic impairment were observed. Furthermore, no serious skin lesions were observed over 48 hours using the Arctic Sun 2000^®^, only a physiological hyperaemia of the covered skin areas with a fast regression within minutes after removing the energy transfer pads from the skin was seen. Other potential methods of quickly lowering body temperature include manual cooling and invasive procedures such as continuous veno-venous haemofiltration (CVVH). During the last few years novel devices originally designed for the delivery of mild therapeutic hypothermia in survivors of cardiac arrest have become available. This case presentation demonstrates that the use of a non-invasive cooling device is safe and efficient in the management of severe hyperthermia and has several advantages compared to other potential methods of temperature management. Invasive cooling by CVVH necessitates the immediate creation of vascular access which would not only pose an additional risk especially in a potentially dehydrated patient but would also account for a certain time delay before the start of cooling therapy. Methods of manual cooling are less reliable because they lack the automated temperature monitoring and feedback regulation and, in addition, manual cooling will probably be much more time consuming with regard to human resources. We therefore believe that in these rare cases of excessive hyperthermia, device-assisted non-invasive cooling should be a treatment of choice wherever available.

**Table 2:** Differential diagnoses with their most common triggers

Disease	Most common trigger
Malignant neuroleptic syndrome	Neuroleptics
Malignant hyperthermia	All volatile anaesthetics and depolarizing neuromuscular blocking agents
Serotonin syndrome	Amphetamine
Heatstroke	General heat accumulation
Sunstroke	General solar radiation
Sepsis	Infection focus
Convulsion	Epilepsy

Since the efficacy of non-invasive cooling became evident very quickly there was no need to institute further therapeutic regimes such as invasive cooling via extracorporeal circulation. After the patient's haemodynamic condition had been stabilized, extracorporeal renal support became necessary later because of acute renal failure probably due to rhabdomyolysis. Dantrolene was discussed as a further possible option in case symptomatic therapy did not improve the patient's condition. However the use of Dantrolene is limited if the liver function is reduced [3]. In the case presented the patient already showed elevated liver enzymes on admission. In the literature a wait-and-see-approach is recommended with symptomatic therapy to avoid the temptation to rush into drug treatment [2]. The decision to use or not use a specific drug therapy should be made in the first 72 hours if the patient's condition does not improve with supportive symptomatic therapy. Symptoms were rapidly reversed by effective cooling and standard supportive care. Residual impairment was mild although typical for excessive hyperthermia. There is a number of reports which describe the finding of isolated cerebellar lesions demonstrated by MRI scan after a condition of hyperthermia [4],5,[7]. Two mechanisms for these damages are discussed: on the one hand, a direct thermal injury to the Purkinje cells and, on the other hand, a cerebellar ischaemia caused by increased intracranial pressure combined with autonomic dysfunction [4,7].

Taking these reports into consideration, harmful effects on brain structures due to hyperthermia and, therefore, a direct cause for the isolated cerebellar lesions seems possible in this case presentation.

## Conclusion

The non-invasive cooling device posed a very efficient and safe solution to treat excessive hyperthermia. Neither serious local nor systemic side effects in our setting were observed. In this rare case of excessive hyperthermia caused by neuroleptic malignant syndrome, the fast cooling therapy probably reduced residual impairment and may have even been lifesaving. We therefore believe that the use of the novel generation of commercially available non-invasive cooling devices can be strongly recommended in cases of severe hyperthermia.

## Abbreviations

NMS: neuroleptic malignant syndrome; ICU: intensive care unit; GCS: Glasgow coma scale; CT: computed tomography; MRI: magnetic resonance imaging; ILCOR: International Liaison Committee on Resuscitation; CVVH: continuous veno-venous haemofiltration.

## Consent

Written informed consent was obtained from the patient for publication of this case report and accompanying images. A copy of the written consent is available for review by the Editor-in-Chief of this journal.

## Competing interests

The authors declare that they have no competing interests.

## Authors' contributions

CS, RG and AK mainly treated the patient during hospital stay and wrote the case report. LN, JCS and DH did the analysis of already published data dealing with severe hyperthermia, whereas FM did the proofreading of the manuscript and revised it critically for important intellectual content.
